# Metabolic and Biosynthetic Diversity in Marine Myxobacteria

**DOI:** 10.3390/md16090314

**Published:** 2018-09-05

**Authors:** Katja Gemperlein, Nestor Zaburannyi, Ronald Garcia, James J. La Clair, Rolf Müller

**Affiliations:** 1 Department of Microbial Natural Products (MINS), Helmholtz Institute for Pharmaceutical Research Saarland (HIPS)—Helmholtz Centre for Infection Research (HZI), Campus E8 1, 66123 Saarbrücken, Germany; katja.gemperlein@helmholtz-hips.de (K.G.); nestor.zaburannyi@helmholtz-hips.de (N.Z.); ronald.garcia@helmholtz-hips.de (R.G.); 2 German Center for Infection Research (DZIF), Partner site Hannover-Braunschweig, Inhoffenstr. 7, 38124 Braunschweig, Germany; 3 Xenobe Research Institute, P.O. Box 3052, San Diego, CA 92163-1052, USA; 4 Department of Pharmacy, Saarland University, Campus E8 1, 66123 Saarbrücken, Germany

**Keywords:** marine myxobacteria, natural products, secondary metabolism, biosynthesis, genomics, diversity

## Abstract

Prior to 2005, the vast majority of characterized myxobacteria were obtained from terrestrial habitats. Since then, several species of halotolerant and even obligate marine myxobacteria have been described. Chemical analyses of extracts from these organisms have confirmed their ability to produce secondary metabolites with unique chemical scaffolds. Indeed, new genera of marine-derived myxobacteria, particularly *Enhygromyxa*, have been shown to produce novel chemical scaffolds that differ from those observed in soil myxobacteria. Further studies have shown that marine sponges and terrestrial myxobacteria are capable of producing similar or even identical secondary metabolites, suggesting that myxobacterial symbionts may have been the true producers. Recent in silico analysis of the genome sequences available from six marine myxobacteria disclosed a remarkably versatile biosynthetic potential. With access to ever-advancing tools for small molecule and genetic evaluation, these studies suggest a bright future for expeditions into this yet untapped resource for secondary metabolites.

## 1. Introduction

Myxobacteria are Gram-negative bacteria commonly known for their complex multicellular development, large genomes, and production of secondary metabolites. Myxobacterial diversity, which was once thought to be exclusive to terrestrial habitats, has recently been expanded to environments containing high salinity. Within the last two decades, the discovery of halotolerant and obligate marine myxobacteria has resulted in the classification and validation of four new genera, namely *Enhygromyxa*, *Haliangium*, *Plesiocystis*, and *Pseudenhygromyxa* [1,2,3,4,5,6]. To date, each of the isolated and cultivated marine-estuarine-derived myxobacteria occupies the *Nannocystineae* suborder. Many of these isolates are regarded as obligate halophile, requiring up to 2–6% (*w*/*v*) salt for growth [2,7]. Some isolates belonging to the *Cystobacterineae* suborder have also been isolated from sea and brackish sediments. While found in marine ecosystems, these species are only regarded as halotolerant and include strains of *Myxococcus fulvus* HW-1 and *Nannocystis pusilla* B150 [8,9]. Interestingly, the number of species of myxobacteria isolated from saline environments has expanded over the years and has partly led to the discovery of new taxa, as illustrated by *Paraliomyxa miuraensis* SMH-27-4 [10]. Among these marine isolates, *Enhygromyxa* is the most commonly isolated genus based on over 20 16S rRNA gene sequences currently available in public databases.

Comparable to terrestrial programs, marine myxobacteria discovery begins with isolation and identification of new taxonomic groups. This is then followed by characterization of their secondary metabolites. Evidence collected to date indicates that marine myxobacteria produce imporant bioactive leads. Recent evidence suggests that the microbial strains belonging to distant taxa typically offer the greatest metabolic novelty and have hence been prioritized for their small molecule studies [11]. Marine-derived myxobacteria fit right in line with this model as extracts from the recently discovered species have delivered scaffolds that differ from that obtained from soil myxobacteria. To date, eight basic families of secondary metabolites including haliangicin [12], haliamide [13], miuraenamides [10], phenylnannolones [14], salimabromide [15], enhygrolides, salimyxins [16,17], and enhygromic acid [17] have been isolated from saline-tolerant or saline-obligate myxobacteria.

The fact that methods are now becoming available for laboratory-based culturing [18], and that new tools are available to reduce the compound requirements for structure elucidation [19], has generated a rich forum for future exploration of marine myxobacteria as a source for biologically relevant secondary metabolites. However, there is a need for methods that allow a reduction of the lengthy periods normally required for microbial growth. Furthermore, there is a need for techniques that enable access to a wide variety of hard-to-cultivate marine strains. Having said that, recent analyses of the environmental samples have shown an enormous diversity of myxobacteria in the marine, estuarine, and other saline ecological niches [20,21]. These studies, along with genome sequence data, clearly identify an enormous diversity of marine-derived myxobacteria. Given the large size of these organisms’ genomes (ranging from 9 to 12.5 Mbp in known isolates [22]), it is likely that they contain a variety of complex, encoded biosynthetic gene clusters. Understanding these synthases and their products will provide new access to secondary metabolites and begin the process of defining a vital new resource for natural product drug discovery.

## 2. Marine-Derived Myxobacterial Natural Products

**Figure 1:**
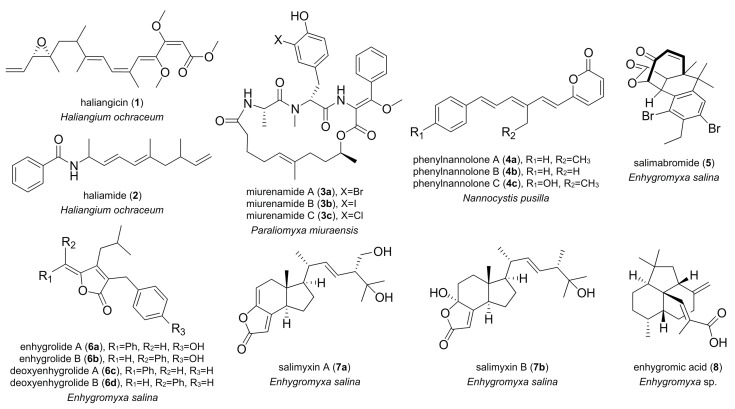
A selection of natural products isolated from extracts of marine myxobacteria.

Only a few metabolites from marine-derived myxobacteria have been published to date (Figure 1). The first myxobacterial compound of true marine origin was haliangicin (**1**), produced by *Haliangium ochraceum* SMP-2 [5] (originally *Haliangium luteum* AJ-13395), which was isolated from a seaweed specimen collected in Japan [12,23]. This polyene features a conjugated tetraene moiety, together with a β-methoxyacrylate [12]. Biological activity studies indicated that haliangicin (**1**) specifically inhibits the electron transport within complex III of the respiratory chain of filamentous fungi [23]. *H. ochraceum* SMP-2 was also shown to synthesize haliamide (**2**), a polyketide-nonribosomal peptide hybrid [13]. Compound **2** demonstrates cytotoxicity against the HeLa S3 tumor cell line [13]. Miuraenamides **3a**–**3c** represent cyclic depsipeptides with a β-methoxyacrylate moiety. They were isolated from *Paraliomyxa miuraensis* SMH-27-4, a slightly halophilic myxobacterium discovered in Japan [10,24]. The major metabolite, miuraenamide A (**3**), was found to exhibit antifungal activity, especially against the phytopathogenic oomycete *Phytophthora capsici*, by inhibiting NADH oxidases [10]. Moreover, miuraenamide A (**3**) was shown to significantly change the morphology of the cytoplasm and the nucleus of HeLa cells by stabilizing actin filaments [25,26]. In other studies, phenylnannolones A–C (**4a**–**4c**) were isolated from extracts of the halotolerant myxobacterium *Nannocystis pusilla* B150 (originally described as *Nannocystis exedens* strain 150), which was isolated from an intertidal region off Crete [8,14]. These compounds contain unusual structural elements, as they are composed of an ethyl-substituted polyene chain linked to a pyrone moiety and a phenyl ring. Phenylnannolone A (**4a**) was shown to have inhibitory activity towards P-glycoprotein and was shown to reverse daunorubicin resistance in cancer cell lines [14].

Strikingly, the majority of marine-derived myxobacterial natural products known so far derive from the obligate marine *Enhygromyxa* species. Up to now, five different compound classes have been identified from this genus. These include salimabromide (**5**) [15], enhygrolides **6a**–**6b** and salimyxins **7a**–**7b** [16], enhygromic acid (**8**) and deoxyenhygrolides **6c**–**6d** [17]. Salimabromide (**5**) was the first secondary metabolite discovered in the genus *Enhygromyxa* [15]. The molecule was isolated from strain *Enhygromyxa salina* SWB007 obtained from a marine mud sample. Structure elucidation studies indicated that it contained a new tetracyclic carbon skeleton comprising a brominated benzene ring, a furano lactone residue, and a cyclohexane ring bridged by a seven-membered cyclic moiety. Antimicrobial testing of salimabromide revealed a moderate antibiotic activity against the Gram-positive bacterium *Arthrobacter* sp. [15]. Besides salimabromide, strain *Enhygromyxa salina* SWB007 produces enhygrolides A (**6a**) and B (**6b**), containing a α/β-unsaturated γ-lactone moiety that is 2,4-substituted with benzyl and benzylidene rings [16]. The closely related marine strain *Enhygromyxa salina* SWB005 biosynthesizes salimyxins A (**7a**) and B (**7b**), representing structurally most unusual, degraded tricyclic sterols [16]. Enhygrolide A (**6a**) and salimyxin B (**7b**) were both shown to inhibit the growth of *Arthrobacter* sp. [16].

In 2017, a new species of *Enhygromyxa*, *Enhygromyxa* sp. SNB-1, was isolated from coastal sand obtained from a beach in Kashiwazaki, Japan. This strain was identified as a producer of the secondary metabolites enhygromic acid (**8**) and deoxyenhygrolides A (**6c**) and B (**6d**) [17]. Structural studies indicate that **8** contains a unique decahydroacenaphthylene motif connected with a α-methylacrylic acid. It exhibits cytotoxic activity against B16 melanoma cells and enhances neurite outgrowth of PC12 cells [17]. While γ-alkylidenebutenolides deoxyenhygrolides **6c**–**6d** are structurally related to the bioactive enhygrolides **6a**–**6b** [16], cytotoxicity and antibiotic screening efforts indicated that deoxyenhygrolides **6c**–**6d** remain inactive [17]. In addition to secondary metabolites, *Enhygromyxa* salina SHK-1^T^ was shown to produce the long-chain polyunsaturated fatty acids (LC-PUFAs) arachidonic acid (20:4, *n*-6) and eicosapentaenoic acid (20:5, *n*-3) [3,27]. Comparable arachidonic acid production of marine origin was also observed in the myxobacteria *Plesiocystis pacifica* SIR-1^T^, which was isolated from Japanese coastal saline environments [2], and *Pseudenhygromyxa salsuginis* SYR-2^T^, which was obtained from an estuarine marsh in Japan [1].

## 3. Semblance between Terrestrial Myxobacterial and Marine Natural Products

**Figure 2:**
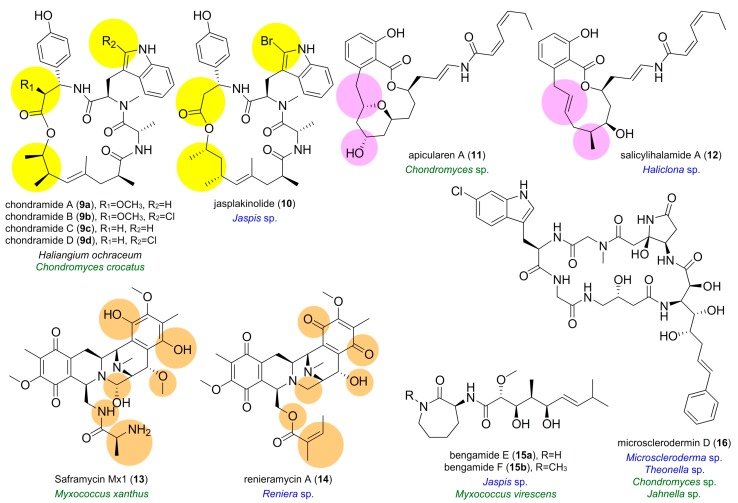
Similarity between natural products isolated from myxobacteria and marine sponges. Analogs of natural products isolated from marine sponges have been observed in cultures of terrestrial myxobacteria. Salient examples include the comparison of chondramides A–D (**9a**–**d**) to jasplakinolide (**10**), apicularen A (**11**) to salicylihalamide A (**12**), and saframycin Mx1 (**13**) to renieramycin A (**14**). Similarly, sponge natural products including bengamides E–F (**15a**–**b**) and microsclerodermin D (**16**) have been obtained from terrestrial myxobacteria. Highlighted regions provide structural comparisons. Species names are given by terrestrial myxobacteria (in green) and sponges (in blue).

Among marine sources, sponges have proven to be highly prolific in their delivery of natural products [28]. Interestingly, several terrestrial myxobacteria are capable of producing secondary metabolites, which are similar or identical to those isolated from marine sponges (Figure 2). For instance, chondramides **9a**–**9d**, produced by the terrestrial myxobacterium *Chondromyces crocatus* [29], show a remarkable structural similarity to jasplakinolide (jaspamide) (**10**) isolated from the marine sponge *Jaspis* sp. [30,31]. Both of these cyclodepsipeptides exhibit antiproliferative activity against carcinoma cell lines by inducing actin polymerization [32].

In other studies, the structure of macrolide apicularen A (**11**), synthesized by species of the terrestrial myxobacterial genus *Chondromyces* [33], was shown to be closely related to that of salicylihalamide A (**12**) obtained from the marine sponge *Haliclona* sp. [34]. Both **11** and **12** were identified as potent inhibitors of the mammalian V-ATPase [35,36]. Similarly, tetrahydroisoquinoline metabolites saframycin Mx1 (**13**) and renieramycin A (**14**) were isolated from the terrestrial myxobacterium *Myxococcus xanthus* and the marine sponge *Reniera* sp., respectively [37,38]. Preliminary screening efforts indicated that saframycin Mx1 (**13**) display both antitumor and antimicrobial activity [39].

As illustrated in Figure 2, the same natural products have been observed in extracts of marine sponges and terrestrial myxobacteria. For example, the antiproliferative and anti-inflammatory bengamides E (**15a**) and B (**15b**) were first isolated from a marine sponge of the genus *Jaspis* [40,41,42]. Astonishingly, the same compounds are also produced by the terrestrial myxobacterium *Myxococcus virescens* [43,44]. The antifungal cyclic hexapeptide microsclerodermin D (**16**) is another example of the same compound obtained from marine sponges *Microscleroderma* sp. and *Theonella* sp. and terrestrial myxobacteria including *Chondromyces* sp. and *Jahnella* sp. [45,46]. This similarity in chemical structures suggests that marine myxobacterial symbionts may be the obligate source for these materials. Interestingly, studies of metagenomes of marine sponges have provided evidence that myxobacteria may even exist as sponge symbionts [47,48]. Furthermore, the finding that several bioactive compounds derived from marine sponges correspond with secondary metabolites produced by terrestrial myxobacteria is particularly intriguing as their terrestrial counterparts can be cultured in the laboratory.

## 4. Biosynthesis of Marine Myxobacterial Natural Products

### 4.1. Haliangicin

The structure of haliangicin (**1**) indicates that its biosynthetic origin arises from a polyketide synthase (PKS) and subsequent methylation and epoxidation tailoring enzymes. Feeding studies with ^13^C-labelled precursors disclosed the biosynthetic building blocks of **1** [49]. Incorporation of two acetate units, one acetate-derived β-methyl branch, four propionate units, three *S*-adenosylmethionine-derived *O*-methyl groups, and a glycerol-derived two-carbon unit bearing vicinal oxygen atoms was demonstrated. In order to identify and to heterologously express the corresponding haliangicin (*hli*) biosynthetic gene cluster, a cosmid library of *H. ochraceum* SMP-2 was constructed and screened with probes containing sequences of ketosynthases (KS) from terrestrial myxobacteria [49]. The *hli* gene cluster, spanning 48 kb with 21 coding sequences, could be identified and characterized in detail. Five type I PKS genes (*hliF*, *G*, *P*, *S*, *T*), a β-methyl branching cassette (*hliL*, *M*, *N*, *O*, *C*), a methoxymalonyl-acyl carrier protein (ACP) cassette (*hliQ*, *H*, *I*, *J*, *K*), an *O*-methyltransferase gene (*hliD*), a metallo-β-lactamase-type thioesterase gene (*hliE*), an acyl-CoA dehydrogenase gene (*hliR*), an epoxidase gene (*hliU*), and two ABC transporter genes (*hliA*, *B*) were annotated.

**Figure 3:**
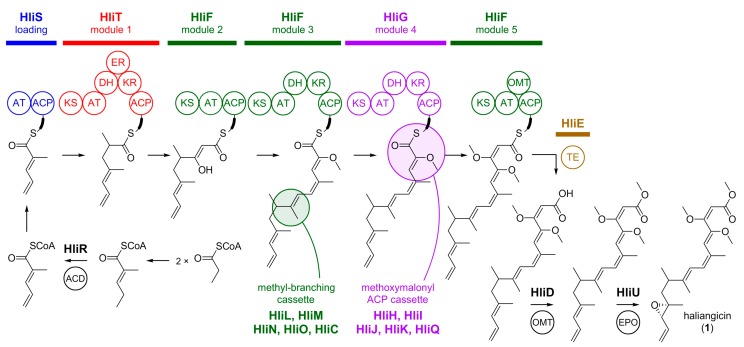
Haliangicin biosynthesis in *Haliangium ochraceum*. ACD = acyl-CoA dehydrogenase, AT = acyltransferase, ACP = acyl carrier protein, KS = ketosynthases, DH = dehydratase, ER = enoyl reductase, KR = ketoreductase, OMT = *O*-methyltransferase, TE = metallo-β-lactamase-type thioesterase, EPO = epoxidase. AT, ACP, KS, DH, ER, KR and TE are enzymes commonly observed in PKSs; OMT and EPO are tailoring enzymes.

Heterologous expression in the potent and reliable myxobacterial host *Myxococcus xanthus* [50,51,52] led to a tenfold higher production of **1** compared to the native producer, thereby proving the identity of the defined *hli* gene cluster [49]. The proposed biosynthesis of **1** is depicted in Figure 3. It begins with the diene molecule 2-methylpent-2,4-dienoyl-CoA, synthesized by γ,δ-dehydrogenation of 2-methylpent-2-enoyl-CoA by an acyl-CoA dehydrogenase to form a terminal olefin [49]. The resulting starter unit is then loaded onto the PKS loading module, and synthesis of the polyketide backbone of **1** begins with sequential elongation with methylmalonate and malonate by modules 1 and 2, respectively. After the elongation step in module 2, the β-methyl branch at position 9 likely arises by a methyl-branching cassette [53,54], and additional methylmalonate unit is incorporated by module 3. The methoxymalonyl-ACP cassette [55,56] is assumed to produce the glycolate extender unit for module 4. After the final extension with malonate in module 5, we propose that the polyketide scaffold is released by a metallo-β-lactamase-type thioesterase [57,58], followed by a post-assembly *O*-methylation at the carboxyl terminus by HliD and epoxidation at the diene terminus by HliU [49].

### 4.2. Haliamide

The structure of haliamide (**2**) suggests that its biosynthetic origin lies in a hybrid PKS and nonribosomal peptide synthetase (NRPS). Feeding studies with various ^13^C-labelled precursors revealed one acetate unit, one acetate-derived terminal methylene unit, two propionate units, one alanine unit, and benzoate as the biosynthetic precursors [13]. A putative haliamide (*hla*) biosynthetic gene cluster was identified by a genome mining approach using the genome sequence of *H. ochraceum* SMP-2 [59]. The putative *hla* gene cluster spans 22 kb and consists of a NRPS/PKS hybrid gene (*hlaA*) and a PKS gene (*hlaB*) [13]. The proposed biosynthesis of **2** begins with benzoyl-CoA. Due to the absence of a PKS loading module, we speculate that the condensation (C) domain of module 1 might catalyze the formation of the amide bond between benzoyl-CoA and the peptidyl carrier protein (PCP)-bound alanine [60]. Synthesis of the polyketide part of **2** is proposed to begin with elongation with malonate by module 2 and proceed with extension with two methylmalonate units by modules 3 and 4. Surprisingly, several essential domains are missing in the PKS modules, such as acyltransferases (AT) in modules 2 and 5, a dehydratase (DH) in module 3, and a ketoreductase (KR) in module 5. It was speculated that the missing functions could be complemented by compatible trans-acting enzymes encoded at other loci in the genome [13]. After the final extension with malonate in module 5, the scaffold may be released by a peculiar decarboxylation, leading to the formation of the terminal olefin of **2**. This chain termination might by catalyzed by the sulfotransferase (ST)-thioesterase (TE) domains encoded at the C-terminus of HlaB [61].

### 4.3. Phennylnannolones

The structure of the phenylnannolones **4a**–**4c** indicates that their biosynthetic origin arises from a PKS. Feeding studies with ^13^C-labelled precursors disclosed three acetate units, one acetate-derived single carbon, one butyrate unit, and a phenylalanine-derived eight-carbon unit as the biosynthetic precursors [14]. A putative phenylnannolone (*phn*) biosynthetic gene cluster was identified by a combination of a genome mining approach using the sequence of *N. pusilla* B150 and a screening of a fosmid library of *N. pusilla* B150 with primers specific for sequences of a TE domain and KS domains [8]. The putative *phn* gene cluster spans 24 kb and consists of a carboxyl transferase gene (*phn1*) and a PKS gene (*phn2*). The proposed biosynthesis of phenylnannolone A (**4a**) begins with cinnamate derived from phenylalanine, which is adenylated by the AMP-dependent ligase/synthetase of the PKS loading module and transferred to the adjacent ACP domain. Synthesis of the polyketide backbone of **4a** may begin in module 1 with elongation with ethylmalonate. We speculate that this building block is synthesized by carboxylation of a butyrate moiety catalyzed by the putative butyryl-CoA carboxylase Phn1 rather than a crotonyl-CoA carboxylase/reductase [62]. Synthesis of the polyketide portion of **4a** is proposed to proceed with extension with three malonate units by modules 2–4. After reduction of the β-keto group by the KR domain and dehydration catalyzed by the DH domain in module 4, a *cis*-double bond is generated. The stereochemistry of this double bond in combination with the keto-enol tautomerism of the unreduced carbonyl group from module 3 is necessary for the TE-catalyzed lactonization to yield the pyrone moiety in **4a**–4**c** [8].

### 4.4. Polyunsaturated Fatty Acids

Several terrestrial myxobacteria belonging to the suborder *Sorangiineae* were found to have the ability for anaerobic polyunsaturated fatty acids (PUFA) biosynthesis via iteratively acting fatty acid synthase (FAS)-like and PKS-like synthases [51]. These multienzyme complexes are encoded by PUFA (*pfa*) biosynthetic gene clusters. Interestingly, *pfa* gene clusters homologous to the oleic acid/linoleic acid-type *pfa* gene cluster from terrestrial *Sorangium cellulosum* strains and to docosapentaenoic acid/docosahexaenoic acid-type *pfa* gene cluster from terrestrial *Aetherobacter* spp. could be identified in the genome of the arachidonic acid-producing marine myxobacterium *Plesiocystis pacifica* SIR-1^T^ (NCBI RefSeq accession code NZ_ABCS00000000.1). Another representative of the suborder *Nannocystineae*, *Enhygromyxa* salina SHK-1^T^, was also shown to produce the LC-PUFAs arachidonic acid and eicosapentaenoic acid [3,27]. Genome mining using the publicly available genome sequences of the closely related strains *E. salina* DSM 15201 (GenBank accession code JMCC00000000.2), *E. salina* SWB005 (GenBank accession code PVNK00000000.1), and *E. salina* SWB007 (GenBank accession code PVNL00000000.1) [22] also revealed the presence of *pfa* gene clusters similar to the known myxobacterial *pfa* clusters in their genomes. While yet unpublished, these studies indicate that marine myxobacteria make use of the same LC-PUFA biosynthetic machinery as terrestrial myxobacteria.

**Figure 4:**
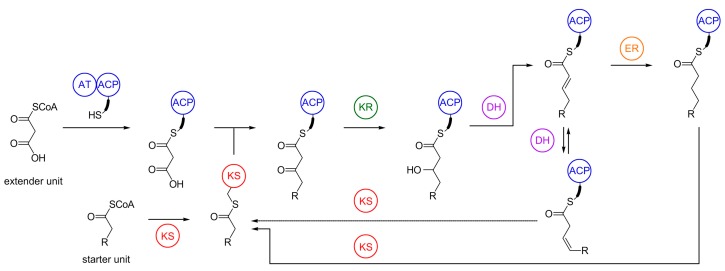
Biosynthesis of long-chain polyunsaturated fatty acids (LC-PUFA) by myxobacterial PUFA synthases. AT = acyltransferase, ACP = acyl carrier protein, KS = ketosynthases, KR = ketoreductase, DH = dehydratase/isomerase, ER = enoyl reductase.

As illustrated in Figure 4, LC-PUFA biosynthesis catalyzed by PUFA synthases proceeds in a similar manner as saturated fatty acids by FASs [63,64,65]. Analogous to the fatty acid assembly line, acetyl-CoA serves as starter molecule, whereas malonyl-CoA, formed by carboxylation of acetyl-CoA using acetyl-CoA carboxylase [66], is required for elongation. The pathway is divided into two phases, initiation and elongation. In the initiation phase, an AT domain transfers the malonyl group of malonyl-CoA to an ACP. The first reaction in the elongation cycle is the decarboxylative Claisen condensation of malonyl-ACP with an acetyl group or the growing acyl chain at the active site cysteine of the KS domain. The β-keto group is then either fully reduced by sequential action of the NADPH-dependent KR domain, the PKS-like DH domain, and the NADPH-dependent enoyl reductase (ER) domain, or it is reduced to the trans-double bond by the KR domain and a FabA-like DH domain and isomerized to a *cis*-double bond. The intermediate then functions as a starter substrate for the next round of elongation with malonyl-ACP until the growing fatty acid chain with methylene-interrupted *cis*-double bonds reaches its final carbon length. Subsequently, the 1-acylglycerol-3-phosphate *O*-acyltransferase (AGPAT) domain, a unique characteristic of myxobacterial PUFA synthases, seems to catalyze the transfer of the synthesized PUFAs from the PUFA synthase into the cellular lipid fractions [51].

## 5. Biosynthesis of Terrestrial Myxobacterial Compounds with Identity to Marine Natural Products

### 5.1. Bengamides

The structure of bengamides E and F (**15a**–**15b**) suggests that they are produced by a hybrid PKS/NRPS. Feeding studies with [2-^13^C]-glycerol revealed the incorporation of two such units [43]. The corresponding begamide (*ben*) biosynthetic gene cluster was identified by a genome mining approach using the sequence of *M. virescens* ST200611 and targeted mutagenesis [43]. The *ben* gene cluster spans 25 kb with nine coding sequences. In detail, three type I PKS genes (*benA-C*), a NRPS gene (*benD*), a methoxymalonyl-ACP cassette (*benE-H*), and a methionine aminopeptidase gene (*benI*) were annotated. Heterologous expression of the *ben* gene cluster in the potent and reliable myxobacterial host *M. xanthus* [50,51,52] led to bengamide production titers comparable to those obtained with the native producer, thereby proving the identity of the defined *ben* gene cluster. The proposed biosynthesis of **15a** and **15b** starts with isobutyrate, which is loaded onto the PKS loading module and extended with malonate by module 1 [43]. The methoxymalonyl-ACP cassette [55,56] is assumed to produce the glycolate extender units for modules 2 and 3. After the final extension with l-lysine in module 4, the scaffold is then released by lactamization catalyzed by the TE domain, resulting in the formation of **15a**. A subsequent post-assembly *N*-methylation at the carboxyl terminus would afford **15b** [43].

### 5.2. Microsclerodermin D

The structure of microsclerodermin D (**16**) indicates that its biosynthetic origin lies in a hybrid PKS/NRPS machinery followed by subsequent post-synthases halogenation by a tailoring enzyme. Feeding studies with isotope-labeled precursors revealed the incorporation of an asparagine unit as well as the α- and β-carbon atoms of phenylalanine [45]. The corresponding microsclerodermin (*msc*) biosynthetic gene cluster was identified by a genome mining approach using the sequences of *Jahnella* sp. Msr9139 and *Sorangium cellulosum* So ce38 (another producer of microsclerodermin derivatives) and targeted mutagenesis of *S. cellulosum* So ce38 [45]. The *msc* gene cluster from *Jahnella* sp. spans 62 kb with 14 coding sequences. In detail, five type I PKS genes (*mscA*, *B*, *C*, *D*, *G*), two NRPS genes (*mscF*, *H*), a NRPS/PKS hybrid gene (*mscI*), a putative amidohydrolase gene (*mscE*), a type II thioesterase gene (*mscJ*), a major facilitator superfamily transporter gene (*mscK*), a tryptophan halogenase gene (*mscL*), a Fe(II)/α-ketoglutarate-dependent oxygenase gene (*mscM*), and a methyltransferase gene (*mscN*) were annotated.

**Figure 5:**
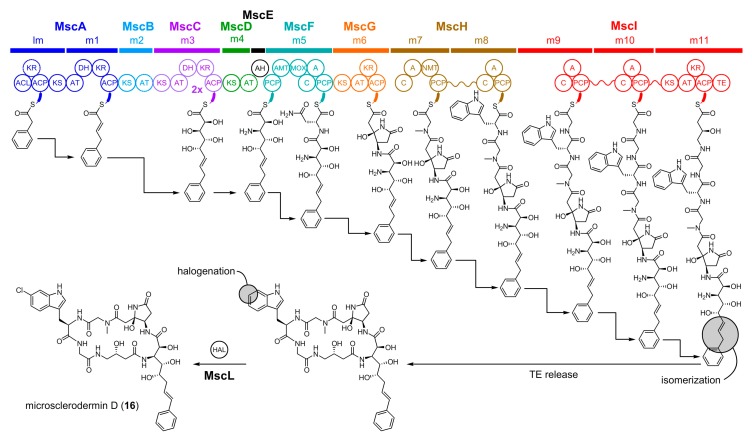
Microsclerodermin D (**16**) biosynthesis in *Jahnella* sp. A = adenylation, ACL = acyl-CoA ligase, ACP = acyl carrier protein, AH = amidohydrolase (putative), AMT = aminotransferase, AT = acyltransferase, C = condensation domain, DH = dehydratase, E = epimerase, HAL = tryptophan halogenase, KR = ketoreductase, KS = ketosynthases, MOX = monooxygenase, NMT = *N*-methyltransferase, PCP = peptidyl carrier protein, and TE = thioesterase. Modules are noted by letter and number as given by m1 for module 1. Curved lines in MscH and MscI denote domains that are covalently attached and are shown separately for ease in depicting the pendant NRPS/PKS chain. AT, ACP, KS, DH, ER, KR, and TE are enzymes commonly observed in PKSs. C, A, PCP, NMT, E and TE are enzymes commonly observed in NRPSs. HAL is a tailoring enzyme. The structural assignment of the microsclerodermins has been a subject of revision [67]. The structure presented for **16** is based on all isolation and chemical synthetic data presented to date.

The proposed biosynthesis of **16** is depicted in Figure 5 and begins with the unusual starter unit phenylacetate [68] on the PKS loading module. Synthesis of the polyketide portion of **16** may proceed in modules 1 and 3 with elongation using one malonate unit and two times 3-hydroxymalonate as extender units in an iterative manner [45]. The PKS-derived unit is forwarded to the first NRPS module, harboring two additional domains showing high similarity to an aminotransferase and a monooxygenase, respectively. Upon chain extension with asparagine, these two domains can oxidize the β-hydroxyl group to the respective β-keto, followed by conversion to a β-amino-group. The resulting intermediate can then undergo cyclization, presumably catalyzed by MscE, to form a rather uncommon pyrrolidone. Thereafter, the biosynthesis continues with a set of chain elongation cycles catalyzed by the forthcoming NRPS and PKS modules corresponding to the observed amino acid and ketide precursors in **16** as evident by domain order and predicted substrate specificity [45]. Final release by a TE and halogenation completes the biosynthesis of **16**.

## 6. Insights into the Metabolic Diversity from the Genomes of Marine Myxobacteria

Our genomic understanding of marine myxobacteria is only beginning to be revealed. At the moment, this knowledge is limited to six genome sequences with varying degrees of completion. Marine myxobacterial strains whose genomes have been made available to date include: *Enhygromyxa salina* DSM 15201 (NCBI GenBank accession code JMCC00000000.2), *Enhygromyxa salina* SWB005 (PVNK00000000.1), *Enhygromyxa salina* SWB007 (PVNL00000000.1), *Haliangium ochraceum* DSM 14365 (NC_013440.1), *Myxococcus fulvus* HW-1 (NC_015711.1), and *Plesiocystis pacifica* SIR-1 (ABCS00000000.1). Various forms of metabolic activity have been investigated within these strains. While early on, these studies have failed to determine if the respective activities are indeed marine specific or if the strains are capable of exerting similar traits when subjected to terrestrial conditions. For example, the presence of a functionally active rubber oxygenase A (*roxA*) gene, whose gene product degrades natural rubber poly-(*cis*-1,4-isoprene) to 2-oxo-4,8-dimethyltrideca-4,8-diene-1-al (ODTD), in the two marine myxobacterial strains *H. ochraceum* DSM 14365 and *M. fulvus* HW-1 hints at a possible bioremediative application for this enzyme [69]. This gene is apparently missing in the remaining genome sequences. Further research on *H. ochraceum* DSM 14365 resulted in finding another rubber-related gene product, RoxB [70] that was capable of degrading the rubber to a mixture of C20 and higher oligo-isoprenoids via the more common c-type heme polyisoprene-degrading system of latex clearing proteins (LCPs) known primarily from Actinobacteria [71,72]. These results indicate *H. ochraceum* DSM 14365 to be a rubber degrader with synergistic or complementary effects across two distinct mechanisms.

Growth in saline habitats requires effective mechanisms to withstand osmotic pressure applied to the cell wall. To combat this stress, bacteria have developed several strategies, mostly focused on the accumulation of either organic compounds called “osmolytes” or inorganic ions within their cells [73]. From the plethora of known organic osmolytes [74], myxobacterial strains studied so far are thought to utilize at least the organic form of osmoregulation, with glutamate, betaine, and hydroxyectoine playing a major role [75]. The combined betaine or hydroxyectoine biosynthetic gene cluster has been found in *E. salina* SWB007 (accession code KU237243) [75]. While the hydroxyectoine component of the combined betaine or hydroxyectoine gene cluster is also encoded as a 6-gene operon in the closely related *E. salina* SWB005 (accession codes for the genes ENSA5_19400-ENSA5_19450), the betaine component is apparently missing. The genome of *H. ochraceum* DSM 14365 also contains a homolog of ectoine synthase (HOCH_RS12805), but the surrounding genes do not share significant similarity that could be identified by Basic Local Alignment Search Tool (BLAST) searching. From the limited knowledge gathered so far, specific osmolytes, such as ectoines, seem to be important only for some of the myxobacteria from saline environments. Occasionally, genes encoding for ectoine-like products could be found even in terrestrial myxobacteria, such as the ectoine synthase in *Stigmatella aurantiaca* DW4/3-1 (STAUR_RS25940), highlighting the difficulty in making a clear division between the two sets of myxobacteria and their lifestyles. On the other hand, osmoregulation in *P. pacifica* SIR-1 seems to be mostly dependent on accumulation of the unmodified amino acids glutamate, glycine, and proline as osmolytes, since experiments have shown no use of betaine, ectoine, or hydroxyectoine as osmotic agents in this bacterium [75].

**Figure 6:**
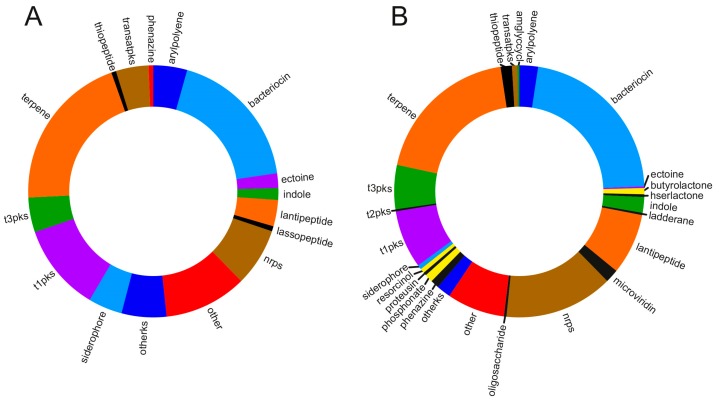
Biosynthetic gene clusters in myxobacterial genomes (**A**) from saline environments (∑ = 181 BGCs) and (**B**) from terrestrial environments (∑ = 467 BGCs). For the computational analysis, we added the following genomic sequences of terrestrial myxobacteria (NCBI reference sequence accession codes in parentheses): *Anaeromyxobacter dehalogenans* 2CP-1 (NC_011891.1), *Anaeromyxobacter dehalogenans* 2CP-C (NC_007760.1), *Anaeromyxobacter* sp. Fw109-5 (NC_009675.1), *Anaeromyxobacter* sp. K (NC_011145.1), *Archangium gephyra* DSM 2261 (NZ_CP011509.1), *Chondromyces crocatus* Cm c5 (NZ_CP012159.1), *Corallococcus coralloides* DSM 2259 (NC_017030.1), *Cystobacter fuscus* DSM 52655 (NZ_CP022098.1), *Melittangium boletus* DSM 14713 (NZ_CP022163.1), *Myxococcus fulvus* 124B02 (NZ_CP006003.1), *Myxococcus hansupus* mixupus (NZ_CP012109.1), *Myxococcus macrosporus* DSM 14697 (NZ_CP022203.1), *Myxococcus stipitatus* DSM 14675 (NC_020126.1), *Myxococcus xanthus* DK 1622 (NC_008095.1), *Sandaracinus amylolyticus* DSM 53668 (NZ_CP011125.1), *Sorangium cellulosum* So ce 56 (NC_010162.1), *Sorangium cellulosum* So0157-2 (NC_021658.1), *Stigmatella aurantiaca* DW4/3-1 (NC_014623.1), *Vulgatibacter incomptus* DSM 27710 (NZ_CP012332.1).

The metabolic landscape of marine myxobacteria is not limited to applications for rubber degradation and osmotic adaptation. According to computational analysis, the marine myxobacterial genomes are nearly on par with terrestrial myxobacteria in terms of potential for secondary metabolite production. As shown in Figure 6, antiSMASH [76] analysis provided an estimate of the potential for natural product biosynthesis encoded within myxobacterial genomes. Using the default program parameters, it was determined that the myxobacterial strains from the saline environments are highly similar to their terrestrial counterparts with regards to the number and content of biosynthetic gene clusters. Concluding from the limited set of genomes used in this comparison, a relative increase in the number of trans-AT-PKS gene clusters and a slightly lower number of NRPS gene clusters are apparent. *M. xanthus* HW-1 attracted our attention particularly because all except for one biosynthetic gene cluster (22 of 23) encoded in its genome sequence could also be found in the related *M. macrosporus* DSM 14697 [77] isolated from soil. This close similarity between these two strains is also reflected in the 16S rRNA gene sequence showing their clustering in the phylogenetic tree [78]. The substantial overlap of biosynthetic gene cluster profiles between two Myxococci raises the question whether such very similar strains can grow under such different conditions, such as soil and saline water, or if such strains are more versatile and can thrive in both ecological niches. Hence, the designation of myxobacterial strains as terrestrial or salt-tolerant requires consideration of multiple traits, particularly if the case remains uncertain.

## 7. Conclusions

To date, a majority of the marine myxobacterial metabolites explored have been found to arise from hybrid type I PKS/NRPS pathways. The biosynthetic pathways of haliangicin [49] and haliamide [13] are encoded within the chromosome of *H. ochraceum* DSM 14365. Although not experimentally confirmed, *M. xanthus* HW-1 is most likely capable of myxochromide A biosynthesis, as it harbors the required core biosynthetic genes encoding proteins that exhibit average protein identity of 85% to the known producers [79]. The genome of *M. xanthus* HW-1 also encodes a DKxanthene biosynthetic gene cluster with a 65% to 85% average protein identity to the known producers *S. aurantiaca* DW4/3-1 [80] and *M. xanthus* DK1622 [81], respectively. Unfortunately, most of the other encoded biosynthetic potential from the analyzed genome sequences remains obscure as only a handful of the respective biosynthetic gene clusters are related to known secondary metabolites.

As most of the myxobacteria isolated to date are of terrestrial origin, the total number of secondary metabolites characterized from marine-derived myxobacteria still remains small. Nevertheless, the short list of natural products obtained from this source already demonstrates novel chemical scaffolds. In silico genome analyses have revealed the great biosynthetic potential of the new genera comprising halophilic or halotolerant myxobacterial species. However, as there is a clear correlation between chemical diversity and phylogenetic distance in myxobacteria in general [11], it is not yet clear whether these marine species do harbor more potential for novel compounds than terrestrial ones. In order to increase the likelihood of identifying novel metabolites with promising bioactivities, it seems encouraging to intensify the exploration of novel genera and families of myxobacteria including marine-derived species. Having said that, marine myxobacteria still offer a rich and vital resource for the future of natural product discovery.
